# Follow-up schedule for initial recurrent hepatocellular carcinoma after ablation based on risk classification

**DOI:** 10.1186/s40644-020-00319-w

**Published:** 2020-07-01

**Authors:** Xuqi Sun, Lingling Li, Ning Lyu, Luwen Mu, Jinfa Lai, Ming Zhao

**Affiliations:** 1 Sun Yat-Sen University Cancer Center, State Key Laboratory of Oncology in South China; Collaborative Innovation Center for Cancer Medicine, Guangzhou, 510060 China; 2 Department of Liver Surgery, Sun Yat-Sen University Cancer Center, Guangzhou, 510060 China; 3 Zhongshan School of Medicine, Sun Yat-Sen University, Guangzhou, 510060 China; 4 Minimally Invasive Interventional Division, Liver Cancer Group, Sun Yat-Sen University Cancer Center, Guangzhou, 510060 China; 5 Department of Vascular Interventional Radiology, Third Affiliated Hospital of Sun Yat-Sen University, Guangzhou, 510060 China

**Keywords:** Ablation, Recurrence, Hepatocellular carcinoma, Follow-up, Survival

## Abstract

**Background:**

To date, no standard follow-up guidelines exist regarding patients receiving ablation for initial recurrent hepatocellular carcinoma (HCC). We aimed to explore whether intensive follow-up could benefit these patients.

**Methods:**

We reviewed the clinical data of patients who received complete ablation for initial HCC recurrence after curative treatments in our institution from January 2005 to June 2017. Risk factors for second recurrence of HCC were identified by univariate and multivariate analyses. Patients were classified into low- and high-risk groups according to the outcome of the classification and regression model. The patients were further categorized into short- (< 3 months) and long-interval (3–6 months) follow-up subgroups based on their surveillance in the first 2 years after complete ablation for initial recurrence. The Kaplan-Meier method with log-rank test was performed to compare the overall survival (OS) based on follow-up intervals in each risk group. We also validated our results by stratifying patients into subgroups with different numbers of risk factors and comparing the OS between patients with different follow-up intervals.

**Results:**

A total of 361 patients were enrolled. The risk factors for secondary recurrence included the Barcelona Clinic Liver Cancer (BCLC) stage at initial recurrence and first recurrence-free survival after curative treatments for primary HCC (*p* < 0.001 and *p* = 0.002). Two risk groups (low and high) were identified. In both the low- and high-risk groups, the OS of patients was not associated with intervals of follow-up (*p* = 0.29 and 0.49). No significant difference was found in the rates of BCLC 0/A stage, tumor location or curative treatments for the second recurrence by different follow-up intervals in each risk group (*p* = 0.34 and 0.87; *p* = 0.69 and 0.97). The same tendency was found in subgroups with 0/1/2 risk factors for secondary recurrence during validation.

**Conclusion:**

The long-interval follow-up did not compromise the survival of patients with complete ablation for initial recurrent HCC.

## Background

Hepatocellular carcinoma (HCC) ranks as the sixth most common malignancy worldwide [1]. Although approximately 80% of the patients suffer from relapse within 5 years after radical treatments for primary HCC, those with recurrences at the Barcelona Clinic Liver Cancer (BCLC) 0/A stage can benefit from resection or ablation [2, 3].Re-resection is often unsuitable for HCC patients with recurrences in consideration of insufficient remnant liver and progressive hepatic dysfunction [4, 5]. Ablation, an effective but less invasive modality, is promising for patients suffering from relapse since most recurrent HCCs are detected by regular follow-up when they are small in size [6, 7]. Cucchetti et al. have reported that radiofrequency ablation is more cost-effective than hepatectomy in HCCs with up to three nodules ≤3 cm [7]. Considering that HCC is prone to recurrence, it makes sense to adopt ablation for initial recurrences to preserve sufficient remnant liver volume and function for potential future treatments.

Compared to patients with primary HCC, patients with initial recurrences can achieve comparable overall survival (OS) after ablation [8, 9]. However, patients with recurrent HCC have shorter disease-free survival than those with primary HCC after ablation [10]. Timely detection and curative treatment of re-recurrence can further improve patient survival and is the purpose of follow-up [11]. Until now, recommendations have varied in different guidelines for surveilling patients after curative treatments and have only been aimed at primary HCC. For the first year after radical therapy for primary HCC, the NCCN guideline recommend regular imaging follow-up every 3–6 months (or 3 months according to the ESMO guideline) [11, 12]. However, no study has specifically demonstrated how to surveil patients after ablation for initial recurrent HCC, who may be at a higher risk for recurrence. Excessively intensive follow-up can increase the waste of medical sources and the anxiety of patients, but delayed follow-up may reduce opportunities for curatively treating re-recurrence.

In this study, we aimed to evaluate whether different follow-up intervals affected patient survival after complete ablation for initial recurrent HCC based on risk classification.

## Methods

### Patient cohort

From January 2005 to June 2017, we reviewed the data of patients with HCC from the database of our institution. The inclusion criteria were curative treatments for primary HCC and complete ablation with the safety margin ≥5 mm for initial recurrence, liver function at Child-Pugh A or B stage, absence of vascular invasion and no extrahepatic metastasis. To ensure the safety margin ≥5 mm, we performed ablation of 1–3 sites per lesion to ensure complete destruction of the tumor and the region 5 mm around it. After ablation, a transverse helical CT scan with the slice thickness of 5 mm was performed. The hypodensity ablated area showed more than 1–2 slides than the initial tumor. In the same slide, the hypodensity ablated area was more than 5 mm larger than the initial tumor, which was calculated by measuring the pre- and post-ablation distance from the corresponding anatomic landmarks. Patients who achieved complete ablation via multiple courses and those who had other primary cancer history were excluded. Finally, we identified 361 patients for this 12-year retrospective study. Our institutional review board approved this study.

### Radiologic and clinical data collection

All patients had contrast-enhanced CT or MRI imaging for primary HCC and initial and second HCC recurrence in our institution. All the lesions were re-evaluated, and controversial nodules were further evaluated and confirmed by another senior radiologist. Recurrent time was the date of initially detecting the recurrent lesion on imaging. Local recurrence was defined as the secondary recurrence occurring in the same segment as the ablated region of initial recurrent HCC [13]. Clinical characteristics reviewed were patients’ sex, age, treatment modality and serum biomarkers. The recorded tumor features included tumor size, number, status of vascular invasion and extrahepatic metastasis. We also evaluated Child-Pugh, albumin bilirubin and BCLC stages for each patient.

**Fig. 1:**
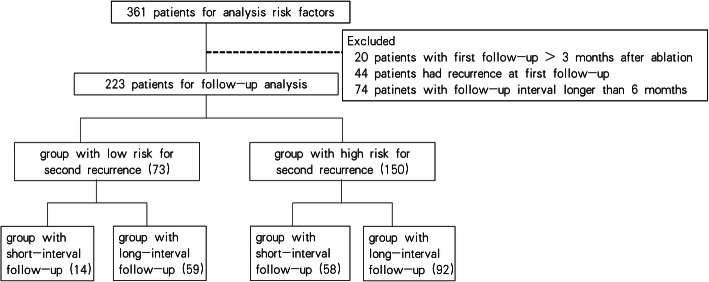
Flowchart of patients included in each analysis

**Fig. 2:**
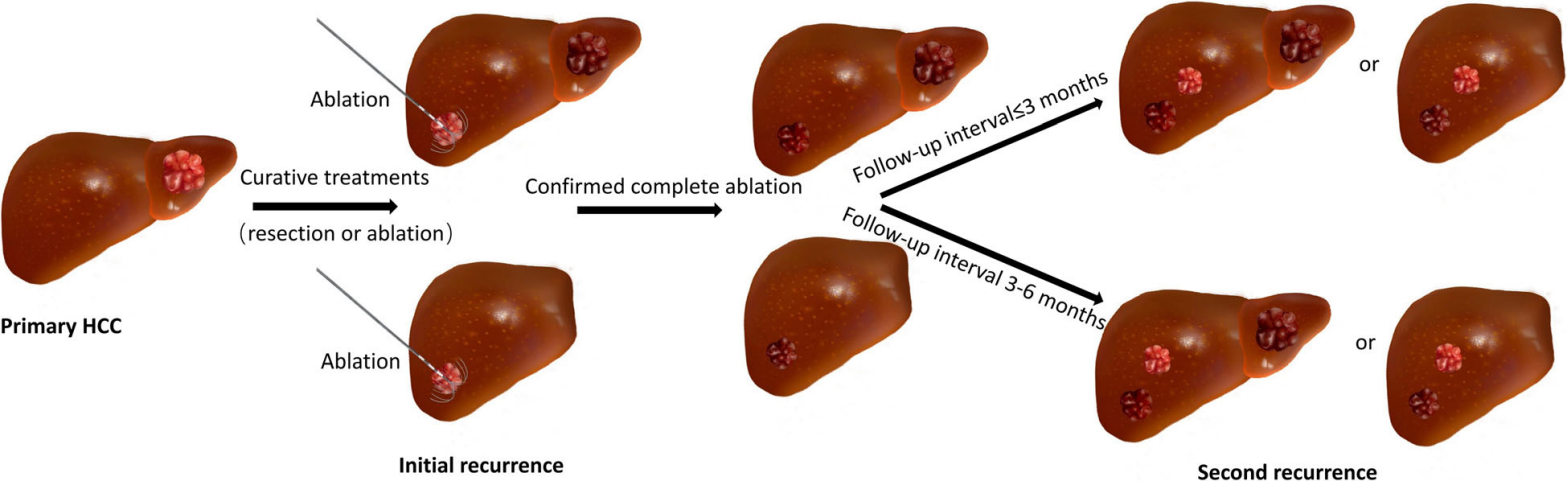
After patients receiving curative treatments for primary HCC, they were treated with ablation for their initial recurrence. All enrolled patients were confirmed to have complete ablation at the first follow-up. Then, patients were followed with different time intervals (≤3 months VS 3–6 months)

Hepatic resection and complete ablation were identified as curative treatment modalities for secondary recurrent HCC. Transcatheter arterial chemoembolization (TACE), molecular targeted treatment and supportive therapy were considered noncurative treatments. This study was censored on July 15, 2019. The study protocol is shown in Fig. 1 and Fig. 2.

### Statistical analysis

To specifically evaluate whether different follow-up regimens affected patients’ prognoses, patients were classified into groups with different risks for secondary recurrence based on the assumption that patients with higher risk of re-recurrence required more intense surveillance. Risk factors were identified by univariate and multivariate analyses, and statistically significant variables were enrolled into a classification and regression model to partition the cohort into groups of different risk rates for secondary recurrence [14]. The Kaplan-Meier (K-M) method with log-rank test was used to evaluate the second recurrence-free survival (RFS) of groups with different risks, and those with no significant difference were merged. Survival curves were plotted by the K-M method for the entire cohort and for groups after risk classification. For validation, we stratified the patients into subgroups based on the number of risk factors for secondary recurrence and evaluated the impact of the follow-up interval on the OS in each group.

For follow-up analysis, we excluded patients with follow-up intervals longer than 6 months in each group after partition, given that patients with HCC history have a higher incidence for subsequent tumors than those only with cirrhosis, while the latter require imaging examination every 6 months. Patients who were followed up > 3 months after ablation or had recurrence during the first follow-up were also excluded. Based on the RFS curve and probability density plot of secondary recurrence for the entire cohort, this study concentrated on the first 2 year of follow-up since 61.2% of the patients suffered re-relapse during this period. Recurrence within 2 years is considered to be most likely from the occult metastasis of the former tumor, while relapse after 2 years is usually tumors of new origin [15]. Therefore, for this study, the optimal follow-up protocol was for the first two post-ablation years. The OS after ablation for initial recurrence was compared based on follow-up intervals in groups with different risk of secondary relapse by the K-M method with the log-rank test. The tumor characteristics and treatment modality at secondary recurrence were compared by Mann-Whitney, Chi-squared and Fisher exact tests. A two-tailed *P* value < 0.05 indicates a significant difference. The density plot, classification and regression model and bootstrapping method were completed with R software version 3.3.2, and the remaining analyses were computed by the IBM SPSS, version 26.0.

## Results

### Patients and risk stratification

**Fig. 3:**
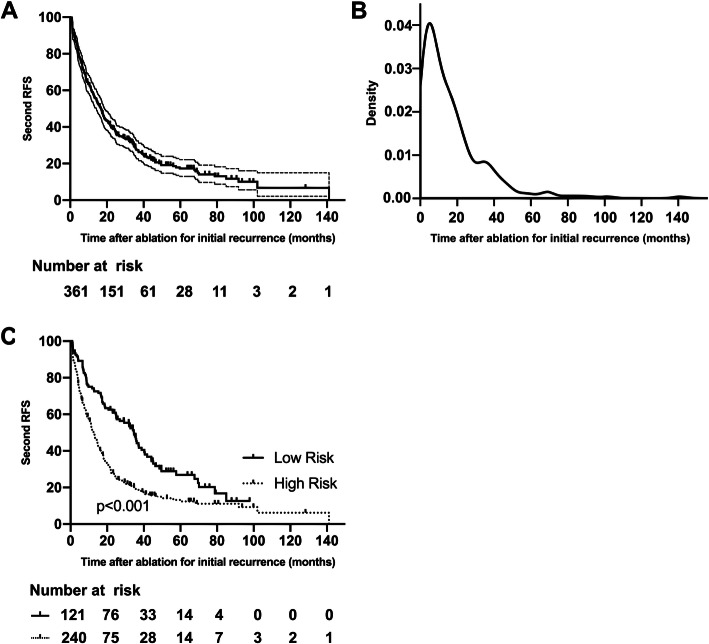
The curves and pattern of second RFS for the 361 patients after receiving ablation for initial recurrent HCC. **a** the second RFS curve with 95% confidence interval; **b** the probability density curve of secondary recurrence; **c** the Kaplan-Meier curves for the low- and high-risk groups stratified by the classification model

The median follow-up time of 361 patients was 36.6 months. Most patients had solitary tumor at initial recurrence with the mean diameter of less than 2 cm (Table 1). In total, 282 (78.1%) patients developed secondary recurrence with 2-, 3-, and 5-year second RFS rates of 38.8, 27.8, and 17.3%, respectively (Fig. 3a). The secondary recurrence most commonly occured in the first 2 years after curative ablation for the initial recurrent HCC (Fig. 3b). Initial recurrence was observed in 347 (96.1%) patients when they were at BCLC 0/A stage, and the rest were diagnosed at BCLC B stage. Most patients only received ablation for radical therapy, while 81 (22.4%) underwent TACE first and then were treated with ablation.

**Table 1:** Baseline Clinical Characteristics of the 361 Patients at Initial Recurrence

Characteristics	Value
Age at initial recurrence, years (IQR)	54.1 (44.0–63.0)
Treatment for primary HCC	
Surgical resection	230 (63.7)
Ablation	131 (36.3)
First recurrence-free survival, months (IQR)	24.9 (7.4–32.7)
Initial recurrent tumor diameter, mm (IQR)	18.4 (12.0–23.0)
Initial recurrent tumor number	
Single	308 (85.3)
Multiple	53 (14.7)
Treatment for initial recurrence	
Ablation	280 (77.6)
TACE + Ablation	81 (22.4)

*TACE* transcatheter arterial chemoembolization, *IQR* interquartile range

**Fig. 4:**
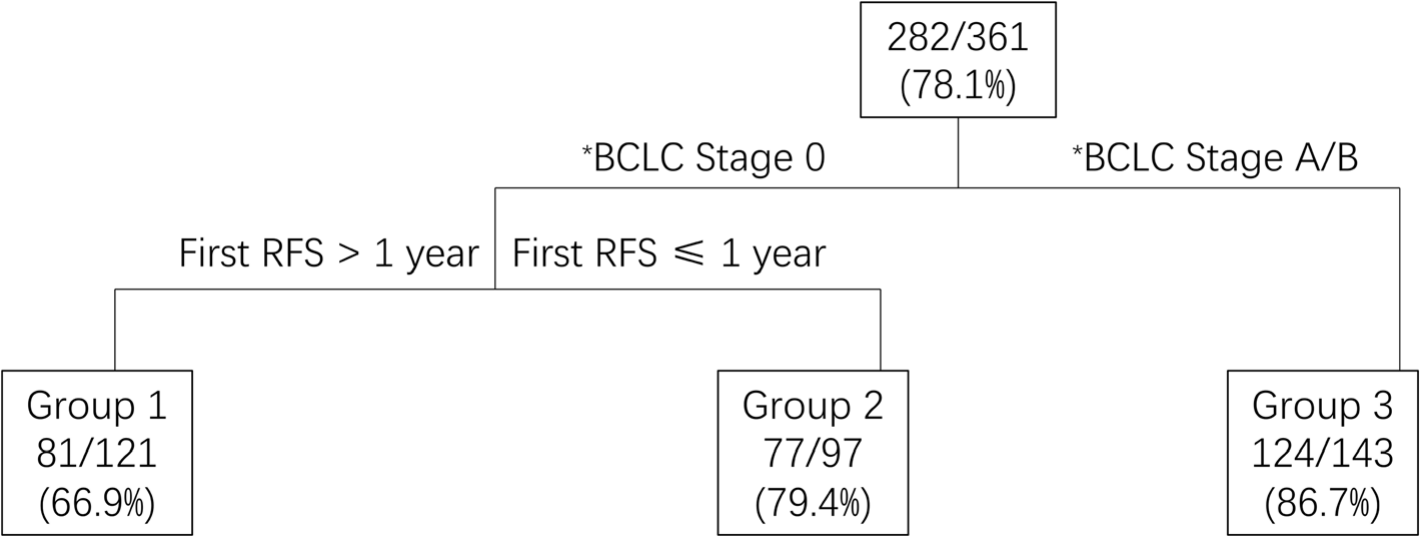
The classification and regression model of the whole cohort. Each terminal node shows the percentage of patients who had secondary recurrence in subgroups

The independent risk factors for secondary recurrence are listed in Table 2. The BCLC stage, serum AST level at initial recurrent HCC, and first RFS after curative treatments for primary HCC were risk factors related to secondary recurrence according to univariate analyses. Multivariate analyses showed that the BCLC stage at initial recurrence and first RFS were related to secondary recurrence (*p* < 0.001 and *p* = 0.002). The classification and regression model categorized the 361 patients into three groups based on their associated risk factors and disease progression. The details are shown in Fig. 4. In the survival analysis, no significant difference in the second RFS and OS between groups 2 and 3 was observed (*p* = 0.760 and 0.239, respectively), and these two groups were merged as the high-risk group, while group 1 was defined as the low-risk group. After merging, the second RFS differed significantly between the two risk groups (*p* < 0.001) (Fig. 3c).

**Table 2:** Univariate and Multivariate Analyses of Risk Factors for Second Recurrence in the 361 Patients

Variables	Univariate	Multivariate	Variable	Univariate	Multivariate
	*P*-value	*P*-value		*P*-value	*P*-value
Factors at primary HCC			**Factors at initial recurrence**		
Male	0.092		Male	0.092	
Age, years	0.121		Age, years	0.222	
pBCLC stage (0/A/B)	0.408		rBCLC stage (0/A/B)	< 0.001*	< 0.001*
HGB, g/L	0.184		HGB, g/L	0.309	
NLR	0.331		NLR	0.632	
PLT, 10^9^/L	0.449		PLT, 10^9^/L	0.295	
WBC, 10^9^/L	0.122		WBC, 10^9^/L	0.140	
AFP, ng/ml (≤200/> 200)	0.207		AFP, ng/ml (≤200/> 200)	0.133	
CA 19–9, U/mL	0.505		CA 19–9, U/mL	0.218	
CEA, ng/mL	0.200		CEA, ng/mL	0.168	
AST, U/L	0.713		AST, U/L	0.022*	0.161
CRE, umol	0.543		CRE, umol	0.339	
ALBI grade (I/II)	0.054		ALBI grade (I/II)	0.111	
Child-Pugh stage (I/II)	0.892		Child-Pugh stage (I/II)	0.385	
Treatment (resection/ablation)	0.075		First RFS, years (≤1/> 1)	0.003*	0.002*
			Pre-TACE (with/without)	0.161	

*pBCLC stage* BCLC stage for primary HCC, *NLR* neutrophil to lymphocyte ratio, *ALBI grade* albumin-bilirubin grade, *rBCLC stage* BCLC stage for initial recurrent HCC, *first RFS* first recurrence-free survival, *pre-TACE* transcatheter arterial chemoembolization before ablation for initial recurrent HCC

### The follow-up for patients

After excluding patients not eligible for follow-up analysis, 223 patients remained. Of them, 73 had low risks of secondary recurrence while 150 had high risk. In each risk group, OS was compared between patients who had short- (≤ 3 months) or long- (3–6 months) follow-up intervals. In total, 14 (19.2%) and 59 (80.8%) patients with low risk received short- and long-interval surveillance, while 58 (38.7%) and 92 (61.3%) patients with high risk received short- and long-interval surveillance. The high-risk group received short-interval follow-up more frequently than the low-risk group (*p* = 0.003). The median times of follow-up intervals were 2.5 months (range: 0.9–3.0 months) and 4.1 months (range: 3.1–6.0 months) in the short- and long-interval groups (*p* < 0.001). The surveillance duration of the short- and long-interval groups was 33.4 and 36.4 months respectively (*p* = 0.91).

### Secondary recurrence and survival of patients with regular surveillance

**Fig. 5:**
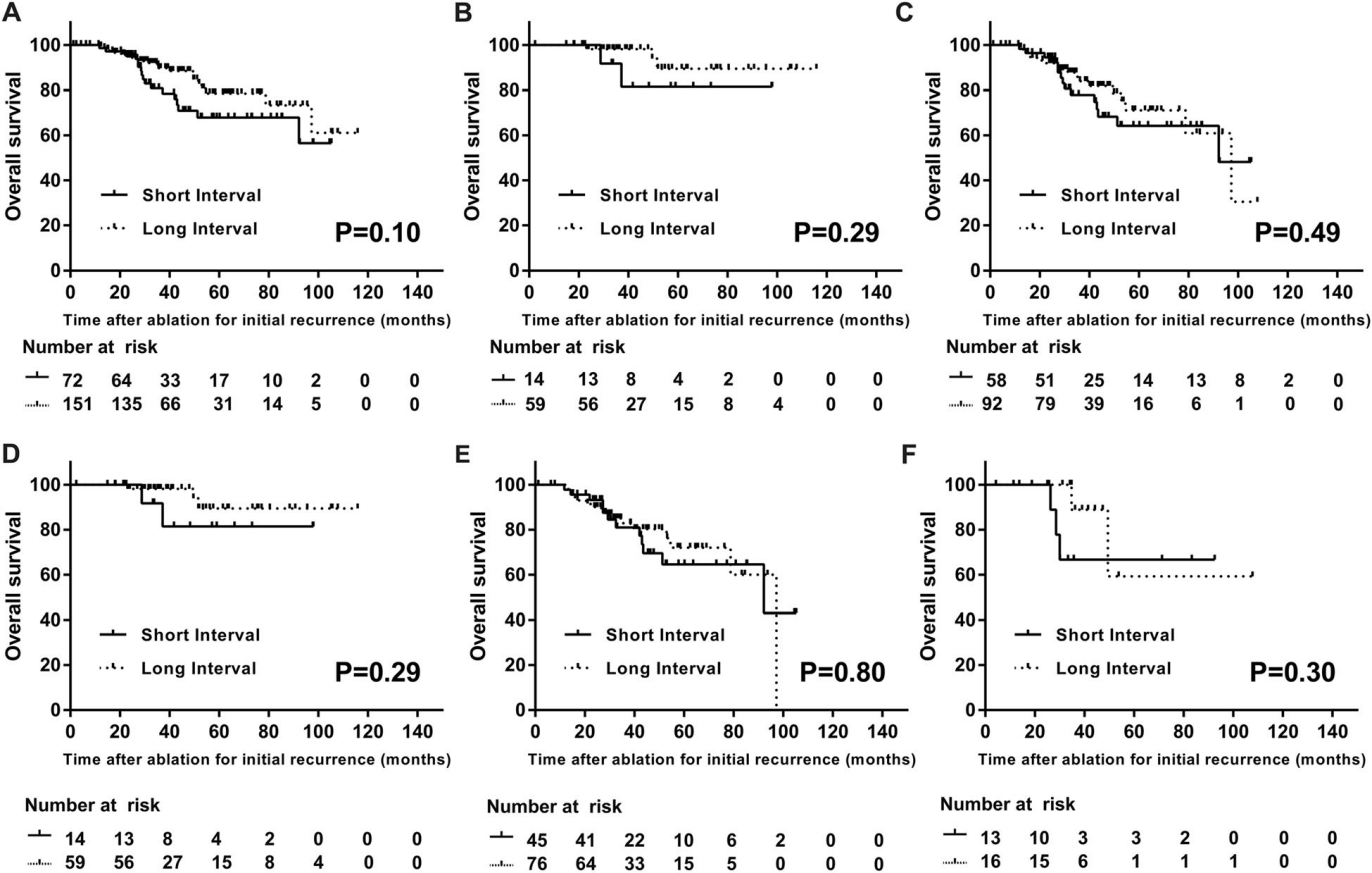
The survival curves for 223 patients under short- and long- interval follow-up with different risks of secondary recurrence. The OS was similar between patients under short- and long-interval follow-up in the whole group (**a**), the low- risk group(**b**) and the high-risk group(**c**). During validation, the OS was also comparable between patients receiving short- and long-interval follow-up in subgroups with 0 (**d**), 1 (**e**) and 2 (**f**) risk factors. The patients with 0 risk factors and those with low risk in the regression model were the same patients

In patients for follow-up analysis, 170 (76.2%) suffered from secondary recurrence, with 46 patients in the low-risk group and 124 patients in the high-risk group. Table 3 shows the characteristics of tumors and treatments at secondary recurrence. The interval of follow-up had no relation with the BCLC stage of secondary recurrence in either the low- or high-risk cohorts. The tumor sizes were also comparable based on different surveillance intervals in each group. The local recurrence rates after ablation for initial recurrence were similar between patients with different follow-up intervals. The distance between secondary recurrent HCC and the ablated area of initial recurrence was not significantly different according to the follow-up interval. The location of secondary recurrence had no significant relation to the follow-up interval. The proportion of patients receiving curative treatments for secondary recurrence was not significantly associated with the follow-up intervals in either group (*p* = 0.686 and 0.967). Accordingly, the survival of patients with different intervals of follow-up was similar (*p* = 0.10). Additionally, OS was similar among patients with different follow-up intervals in both the low- and high-risk groups (*p* = 0.29 and 0.49) (Figs. 5a-c). For patients receiving curative treatments for secondary recurrent HCCs, the OS also had no significant correlation with the follow-up interval in either risk group (*p* = 0.26 and 0.51) (Supplementary Fig. 1).

**Table 3:** Characteristics of Second Recurrent HCCs in the Low- and High- risk Groups

Characteristics	Low Risk			High Risk		
	Short interval	Long interval	*P*-valve	Short interval	Long interval	*P*-value
	*n* = 11	*n* = 35		*n* = 54	*n* = 70	
Tumor diameter (mm)	16.73 ± 10.45	17.79 ± 10.61	0.773	19.03 ± 14.34	21.56 ± 20.66	0.443
SrBCLC stage			0.338			0.872
0/A	11 (100.0)	29 (82.9)		42 (77.8)	56 (80.0)	
B	0 (0.0)	2 (5.7)		7 (13.0)	7 (10.0)	
C	0 (0.0)	4 (11.4)		5 (9.3)	7 (10.0)	
Location			0.640			0.358
Left lobe	2 (18.2)	8 (22.9)		13 (24.1)	13 (18.6)	
Right lobe	8 (72.7)	19 (54.3)		31 (57.4)	41 (58.6)	
Left and right lobes	1 (9.1)	5 (14.3)		7 (13.0)	6 (8.6)	
Metastasis	0 (0.0)	3 (8.6)		3 (5.6)	10 (14.3)	
FrSrDistance (mm)	41.18 ± 40.39	45.70 ± 34.92	0.720	45.29 ± 31.05	49.08 ± 32.37	0.513
Local recurrence			1.000			0.842
Yes	4 (36.4)	11 (31.4)		19 (35.2)	27 (38.6)	
No	7 (63.6)	24 (68.6)		35 (64.8)	43 (61.4)	
Curative treatment			0.686			0.967
Yes	8 (72.7)	21 (60.0)		29 (53.7)	39 (55.7)	
No	3 (27.3)	14 (40.0)		25 (46.3)	31 (44.3)	

*srBCLC stage* BCLC stage for second recurrent HCC*, FrSrDistance* the distance between second recurrent HCC and the ablated area of initial recurrence

### Validating the impact of different follow-up intervals based on the number of risk factors

Of 233 patients qualified for follow-up analysis, 73, 121 and 29 patients had 0, 1 and 2 risk factors for secondary recurrence, respectively. The patients with 0 risk factors and those with low risk in the regression model were the same patients. The characteristics of secondary recurrent HCC were similar in subgroups with 0/1/2 risk factors based on different follow-up intervals. Detailed information is shown in Supplementary Table 1. In each subgroup, the follow-up interval had no significant association with OS (*p* = 0.29, 0.80 and 0.3 respectively) (Figs. 5d-f).

## Discussion

In this study, patients were classified into low- and high-risk groups according to their risk of secondary recurrence. We found that the intervals of follow-up had no significant impact on the survival of patients in either group. The aim of postoperative surveillance is to detect recurrent HCCs in earlier stages, thus, increasing opportunities for curative treatments and improving patient survival outcomes [11]. However, in our study, neither patients with high- nor low-risks benefited from short-interval follow-up in terms of survival, tumor size, BCLC stage, and curative treatment intent rates at secondary recurrence. There are various reasons for the similar outcomes between different follow-up intervals.

In the low-risk group, most patients received curative treatments for secondary recurrences since they were detected at BCLC 0/A stage, where tumors have slow growth rates and less malignant characteristics [16]. Thus, the prognosis had no association with follow-up intervals. In the high-risk group, more cases were diagnosed at BCLC B/C stage for secondary recurrence; thus, TACE and systemic therapy were increasingly given. Short-interval surveillance did not increase opportunities for radical therapy. Patients at high risk for subsequent recurrences had tumors with more malignant biology which limited the survival benefits provided by follow-up [17].

The diameter and number of primary HCC were not significantly related to HCC re-recurrences in this study. Although some studies have demonstrated that the diameter and number of primary HCC had no significant association with the prognosis after initial recurrence, some researchers have reported a significant correlation between the tumor size and survival [18–20]. The above conflicting results might be due to that most of patients enrolled in some studies were at early stage and the difference of tumor size and number may be not sufficient to find a significant correlation.

To date, little information exists on how to follow patients after complete ablation for initial recurrent HCCs. The NCCN guideline recommends multiphasic imaging every 3 to 6 months in the first 2 years after radical treatments, while the ESMO guideline proposes enhanced CT or MRI every 3 months in the first year and every 6 months thereafter [12, 21]. The guidelines have not provided specific suggestions on the surveillance for postoperative patients after initial recurrence.

With the rising incidence of HCC, the number of patients with local recurrences consequently increases. Though ablation can provide comparable prognoses in patients with primary and recurrent HCCs, some studies found that the RFSs are shorter in recurrent ones [8–10]. The 3- and 5-year RFS of complete ablation for recurrent HCCs in our study were similar with those reported by Sun et al., but RFS for recurrent HCCs was shorted than that of primary HCCs [22]. Given that the growth rates of recurrent HCCs is faster than that of primary HCCs after transcatheter arterial chemoembolization, it was considered whether patients need a more intensive follow-up regimen after ablation for initial recurrent HCCs [23]. Hyder et al. reported that ablation associates with more intensive imaging examination during surveillance than hepatectomy but does not confer to better survival benefits [24]. The optimal follow-up time should provide lower medical costs without compromising patient survival. Our study suggested imaging follow-up could be executed every 3 to 6 months in patients after ablation for initial recurrent HCCs in the first 2 years.

Postoperative recurrences frequently occur in the first 2 years after radical treatments for HCC, and postoperative patients have higher risk of subsequent HCC than those only with cirrhosis, so the follow-up interval should not be longer than 6 months, which is the frequency of surveillance recommended for patients at risk of primary HCC [11]. Intensive follow-up can increase social, medical and environmental burden. The cost and radiation dose can be reduced by 25% after extending the surveillance interval from 3 to 4 months by enhanced CT imaging in the first 2 years [25]. Less intensive surveillance can also reduce the use of the enhanced injection, which is associated with allergic reactions and may accumulate in the brain, making its use debatable [26].

Few studies have investigated how postoperative follow-up affects the quality of life of patients with HCC. The GIVIO investigators have reported that intensive surveillance can increase patients’ anxiety in breast cancer [27]. Actually, for both breast and colorectal cancer, intensive follow-up was not found to have a significant reduction in mortality, which is similar with the results in our study [27, 28]. Intensive follow-up may provide limited survival benefits due to the inherent aggressive biologic characteristics of the tumor [29].

Our study has several limitations. First, the model for risk classification was not validated by another independent institution. However, the aim of this model was to identify patients at different risks of HCC re-recurrence but not to predict the prognosis. Second, this study focused on the following protocol in the first 2 years after ablation for initial recurrent HCC since most secondary recurrence occurred during this period, but surveillance thereafter could be evaluated to make a more comprehensive regimen. Third, the retrospective nature of the present study might have potential confounding biases, so multicentered prospective studies are needed for validation.

## Conclusions

In conclusion, comparing with the follow-up interval shorter than 3 months in the first 2 years, a follow-up interval of 3 to 6 months with contrast-enhanced imaging does not compromise the survival of patients receiving ablation for initial recurrent HCCs.

## Supplementary information

**Additional file 1: Supplementary Fig. 1.** The survival curves of patients receiving curative treatments for secondary recurrent HCC. The OS was similar between patients under short- and long-interval follow-up in the whole group (A), the low-risk group (B) and the high-risk group (C). During validation, the OS was also comparable between patients receiving short- and long-interval follow-up in subgroups with 0 (D), 1 (E) and 2 (F) risk factors.

**Additional file 2: Supplement Table 1.** Characteristics of Second Recurrent HCCs in Subgroups with Different Number of Risk Factors

## Data Availability

All data included in this study are available upon request by contact with the corresponding author.

## Abbreviations

HCC: Hepatocellular carcinoma
OS: Overall survival
TACE: Transcatheter arterial chemoembolization
K-M: The Kaplan-Meier
RFS: Recurrence-free survival
