# Supplementary figures and images for: Follow-up schedule for initial recurrent hepatocellular carcinoma after ablation based on risk classification
Source: Cancer Imaging. 2020 Jul 1;20:42. doi: 10.1186/s40644-020-00319-w (PMC7329485; doi:10.1186/s40644-020-00319-w)

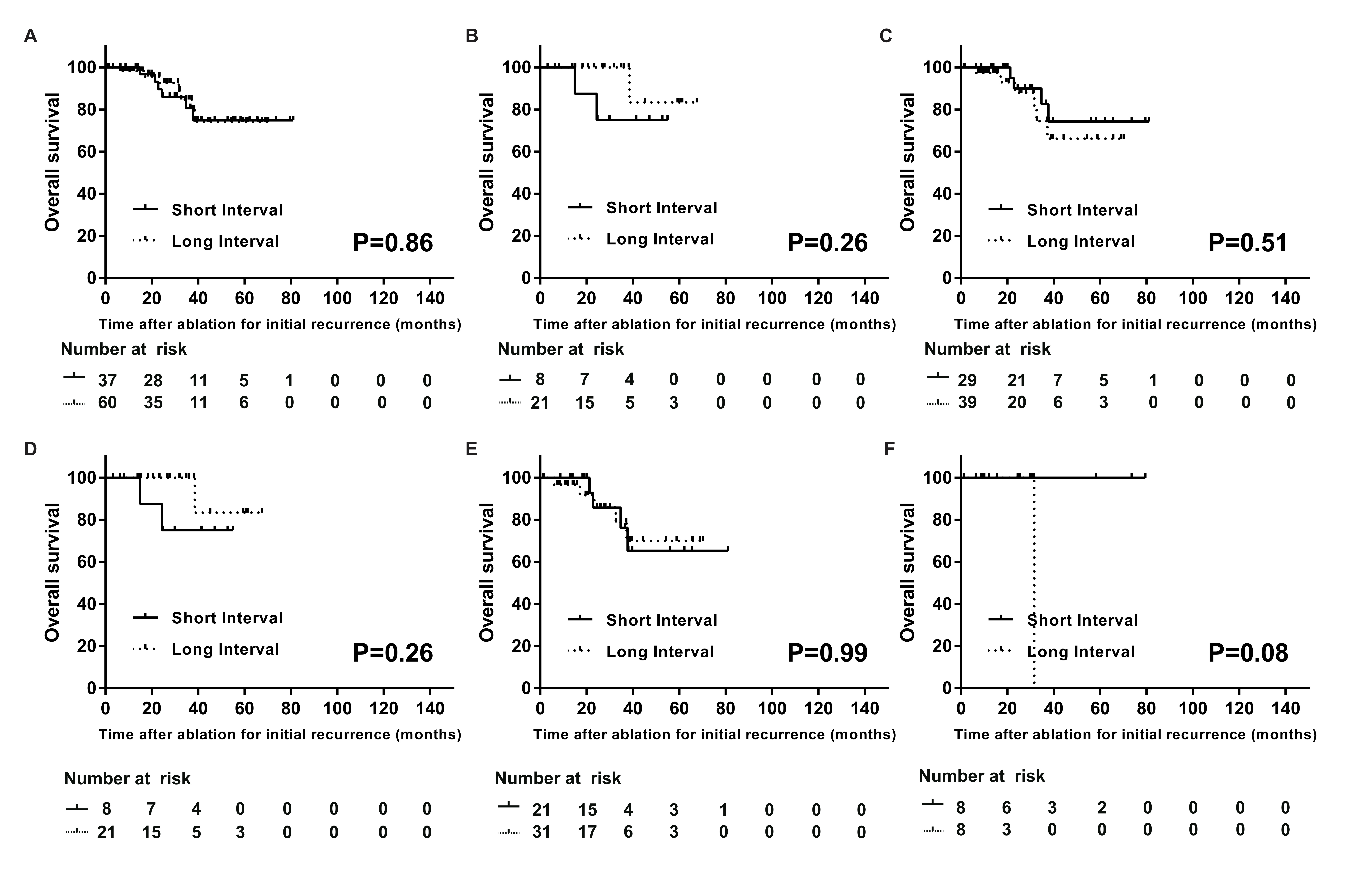

Supplement: Supplementary file 1 — Additional file 1: Supplementary Fig. 1. The survival curves of patients receiving curative treatments for secondary recurrent HCC. The OS was similar between patients under short- and long-interval follow-up in the whole group (A), the low-risk group (B) and the high-risk group (C). During validation, the OS was also comparable between patients receiving short- and long-interval follow-up in subgroups with 0 (D), 1 (E) and 2 (F) risk factors. [file 40644_2020_319_MOESM1_ESM.jpg]
