# Supplementary material for: Follow-up schedule for initial recurrent hepatocellular carcinoma after ablation based on risk classification
Source: Cancer Imaging. 2020 Jul 1;20:42. doi: 10.1186/s40644-020-00319-w (PMC7329485; doi:10.1186/s40644-020-00319-w)
Supplement: Supplementary file 2 — Additional file 2: Supplement Table 1. Characteristics of Second Recurrent HCCs in Subgroups with Different Number of Risk Factors [file 40644_2020_319_MOESM2_ESM.docx]

**Supplement Table 1.** Characteristics of Second Recurrent HCCs in Subgroups with Different Number of Risk Factors

| Characteristics | Group with 0 risk factor | | | Group with 1 risk factor | | | Group with 2 risk factors | | |
| --- | --- | --- | --- | --- | --- | --- | --- | --- | --- |
|  | Short interval | Long interval | P-valve | Short interval | Long interval | P-value | Short interval | Long interval | P-valve |
|  | n=11 | n=35 |  | n=41 | n=56 |  | n=13 | n=14 |  |
| Tumor diameter (mm) | 16.73±10.45 | 17.79±10.61 | 0.773 | 17.94±14.72 | 21.31±18.43 | 0.337 | 22.62±12.28 | 22.56±20.23 | 0.992 |
| SrBCLC stage |  |  | 0.338 |  |  | 0.948 |  |  | 0.568 |
| 0/A | 11 (100.0) | 29 (82.9) |  | 31 (75.6) | 43 (76.8) |  | 11 (84.6) | 13 (92.9) |  |
| B | 0 (0.0) | 2 (5.7) |  | 6 (14.6) | 7 (12.5) |  | 1 (7.7) | 0 (0.0) |  |
| C | 0 (0.0) | 4 (11.4) |  | 4 (9.8) | 6 (10.7) |  | 1 (7.7) | 1 (7.1) |  |
| SrLocation |  |  | 0.640 |  |  | 0.129 |  |  | 0.318 |
| Left lobe | 2 (18.2) | 8 (22.9) |  | 12 (29.3) | 10 (17.9) |  | 1 (7.7) | 3 (21.4) |  |
| Right lobe | 8 (72.7) | 19 (54.3) |  | 23 (56.1) | 31 (55.4) |  | 8 (61.5) | 10 (71.4) |  |
| Both | 1 (9.1) | 5 (14.3) |  | 5 (12.2) | 6 (10.7) |  | 2 (15.4) | 0 (0.0) |  |
| Metastasis | 0 (0.0) | 3 (8.6) |  | 1 (2.4) | 9 (16.1) |  | 2 (15.4) | 1 (7.1) |  |
| FrSrDistance (mm) | 41.18±40.39 | 45.70±34.92 | 0.720 | 46.22±33.59 | 49.39±31.10 | 0.632 | 42.38±22.04 | 47.80±38.34 | 0.660 |
| Local recurrence |  |  | 1.000 |  |  | 1.000 |  |  | 0.802 |
| Yes | 4 (36.4) | 11 (31.4) |  | 15 (36.6) | 21 (37.5) |  | 4 (30.8) | 6 (42.9) |  |
| No | 7 (63.6) | 24 (68.6) |  | 26 (63.4) | 35 (62.5) |  | 9 (69.2) | 8 (57.1) |  |
| Curative treatment |  |  | 0.686 |  |  | 0.843 |  |  | 1.000 |
| Yes | 8 (72.7) | 21 (60.0) |  | 21 (51.2) | 31 (55.4) |  | 8 (61.5) | 8 (57.1) |  |
| No | 3 (27.3) | 14 (40.0) |  | 20 (48.8) | 25 (44.6) |  | 5 (38.5) | 6 (42.9) |  |

srBCLC stage: BCLC stage for second recurrent HCC; FrSrDistance: the distance between second recurrent HCC and the ablated area of initial recurrence.
